# SARS-CoV-2 neurovascular invasion supported by Mendelian randomization

**DOI:** 10.1186/s12967-024-04907-3

**Published:** 2024-01-24

**Authors:** Yiji Pan, Jun Zhang, Tao He

**Affiliations:** 1 Department of Ophthalmology, Renmin Hospital of Wuhan University, 238 Jiefang Road, Wuhan, 430060 Hubei China; 2 Eye Research Center, Renmin Hospital of Wuhan University, Wuhan, China

**Keywords:** COVID-19, Mendelian randomization, Optical coherence tomography, Retina, Genetics

## Abstract

**Background:**

Severe acute respiratory syndrome coronavirus 2 (SARS-CoV-2) is known to affect vessels and nerves and can be easily visualized in the retina. However, the effect of SARS-CoV-2 on retinal morphology remains controversial. In the present research, we applied Mendelian randomization (MR) analysis to estimate the association between SARS-CoV-2 and changes in the thickness of the inner retina.

**Methods:**

Two-sample MR analysis was conducted using summary-level data from 3 open genome-wide association study databases concerning COVID-19 infection (2,942,817 participants) and COVID-19 hospitalization (2,401,372 participants); moreover, the dataset of inner retina thickness, including the macular retinal nerve fiber layer (mRNFL) and macular ganglion cell-inner plexiform layer (mGCIPL), included 31,434 optical coherence tomography (OCT) images derived from healthy UK Biobank participants. All the participants were of European ancestry. The inverse variance weighted (IVW) meta-analysis was used as our primary method. Various complementary MR approaches were established to provide robust causal estimates under different assumptions.

**Results:**

According to our MR analysis, genetically predicted COVID-19 infection was associated with an increased risk of mRNFL and mGCIPL thickness (OR = 1.74, 95% CI 1.20–2.52, P = 3.58 × 10^–3^; OR = 2.43, 95% CI 1.49–3.96, P = 3.6 × 10^–4^). The other MR methods produced consistent results. However, genetically predicted COVID-19 hospitalization did not affect the thickness of the inner retina (OR = 1.11, 95% CI 0.90–1.37, P = 0.32; OR = 1.28, 95% CI 0.88–1.85, P = 0.19).

**Conclusion:**

This work provides the first genetically predictive causal evidence between COVID-19 infection and inner retinal thickness in a European population. These findings will contribute to further understanding of the pathogenesis of COVID-19 and stimulate improvements in treatment modalities.

**Supplementary Information:**

The online version contains supplementary material available at 10.1186/s12967-024-04907-3.

## Introduction

The Mendelian randomization (MR) research design follows the Mendelian genetic law of “parental alleles are randomly assigned to offspring” [1, 2]. MR is an instrumental variable (IV) method that uses single-nucleotide polymorphisms (SNPs) identified by genome-wide association studies (GWAS) as genetic instruments to evaluate the effect of an exposure (e.g., COVID-19 infection) on the risk of an outcome (e.g., inner retinal thickness) [3, 4]. In epidemiological studies, the existence of confounding factors has strongly interfered with the causal inference of exposure and outcome. Since these genetic variants are allocated randomly at conception, MR studies have the advantage of minimizing bias due to confounding factors and reverse causality, with random genotype allocation mimicking intervention allocation in randomized controlled trials (RCTs) [5]. At present, the MR method has been widely used to assess the causal relationships between traits and diseases and between diseases.

Severe acute respiratory syndrome coronavirus 2 (SARS-CoV-2) infection is a multisystem disease. Since the pandemic outbreak of SARS-CoV-2 infection, attention has been widely focused on its pathogenesis and treatment. SARS-CoV-2 can enter cells via the angiotensin-converting enzyme 2 (ACE2) receptor, eliciting an immunological response accompanied by endothelial dysfunction and apoptosis, resulting in micro-thrombotic events [6]. In addition to causing microcirculation disorders, SARS-CoV-2 can infiltrate and affect the central nervous system (CNS) [7, 8].

The retina is an extension of the brain and spinal cord, with comparable damage and immunological responses, and has stable blood flow and maximum metabolic demand among organs in the human body [9]. The macular, as the most sensitive area of vision, has the highest density of retinal ganglion cells (RGCs) and is usually studied as a macular ganglion cell complex (mGCC) via optical coherence tomography (OCT), which consists of the macular retinal nerve fiber layer (mRNFL), macular ganglion cell layer and internal plexiform layer (mGCIPL) [9]. Apparently, both microcirculation and neuronal damage caused by SARS-CoV-2 can affect the macula and cause vision loss. Changes in mGCC thickness measured via OCT can provide additional direct and visible evidence of the effect of SARS-CoV-2 on retinopathy. However, studies of SARS-CoV-2 retinopathy were mostly observational studies and case reports that were controversial due to various confounders from acquired environmental exposure [10, 11].

**Fig. 1:**
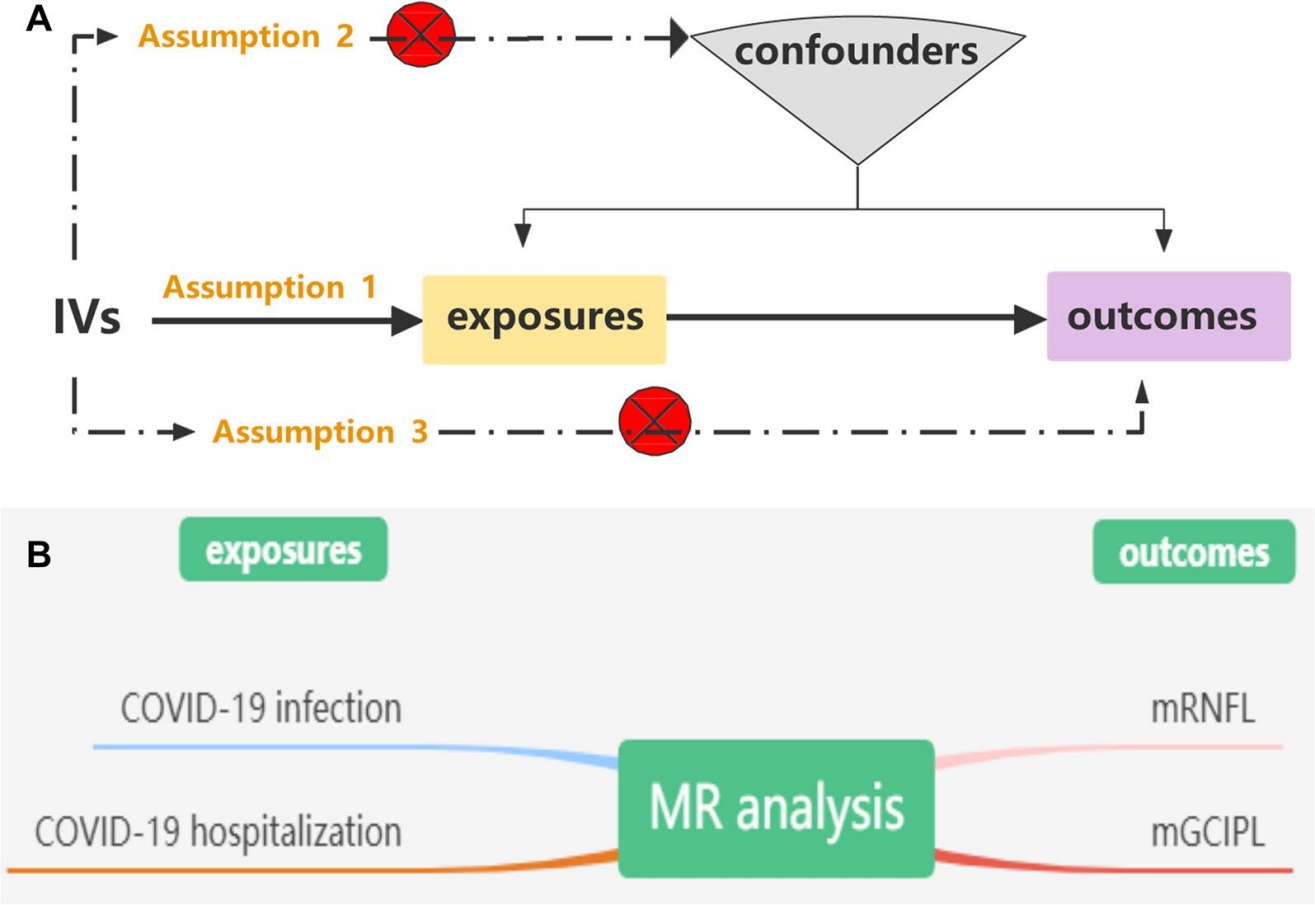
**A** MR analyses depend on three core assumptions. **B** Sketch of the study design. COVID-19 infection and COVID‐19 hospitalization as exposures, mRNFL and mGCIPL as the outcomes

MR analysis can effectively address these issues, as it is a reliable method for identifying causal relationships between diseases [12]. To our knowledge, no MR analysis has been performed to investigate the causal association between genetically predicted COVID-19 and the inner retina. In this study, we performed a two-sample MR analysis to investigate whether SARS-CoV-2 infection was related to mGCC thickness **(**Fig. 1).

## Methods

### Genetic instruments for COVID-19

Three essential model assumptions of MR analysis should be fulfilled to guarantee valid IVs: (1) the selected IVs are strongly associated with exposure factors (COVID-19 infection and hospitalization); (2) the eligible IVs for exposure are not associated with any confounders; and (3) the selected IVs affect only the outcome (thickness of the mRNFL and mGCIPL) exclusively through exposure (COVID-19 infection and hospitalization). A detailed description of the proposed method is provided in Fig. 1.

### GWAS datasets

We conducted a two-sample MR study using publicly available summary statistics from three GWASs. The genetic instruments used for the exposure and outcome datasets were derived from separate and nonoverlapping individual datasets [3]. Two COVID-19 datasets, including COVID-19 infection (2,942,817 participants) and COVID-19 hospitalization (2,401,372 participants) summary association statistics of European ancestry, were obtained from the COVID-19 HGI GWAS round 7(https://www.covid19hg.org/results/r7/) [13]. The COVID-19 infection reflects overall susceptibility to and severity of the virus, whereas COVID-19 hospitalization represents severe patients with hospitalization managements [13]. The mGCC-associated GWAS includes phenotypes of inner retinal morphology derived from OCT images of 31,434 UK Biobank good healthy participants with good vision and no self-reported macular or systemic disease (including diabetes, glaucoma, and neurodegenerative disease) [14, 15]. The average thickness across the macular grid between the two inner retinal layers, the mRNFL and the mGCIPL, was calculated for the left and right eyes [14].

### Statistical analysis

For each exposure phenotype, available SNPs with genome-wide significance (P < 5 × 10^–8^) were selected as IVs. These IVs were further pruned using a clumping r^2^ cutoff of 0.001 within each 10 Mb window using the 1000 Genomes Project Phase 3 as the reference panel. After screening the PhenoScanner database (http://www.phenoscaner.medschl.cam.ac.uk/), SNPs that correlated with confounding factors were manually screened and omitted.

Based on the abovementioned core MR assumptions, MR analyses were conducted via three main complementary methods with the TwoSampleMR package, namely, the inverse variance weighted (IVW), maximum likelihood, and MR‒Egger [16]. The IVW method is a valid analysis assuming the basic premise that all genetic variation is a valid instrumental variable, with strong causality detected. The model is implemented for primary estimation of causal effects, and a P < 0.05 represents a potential causal relationship between the exposure and outcome [12]. MR results are expressed as odds ratios (ORs) with corresponding 95% confidence intervals (CIs) for the outcome risk of corresponding unit changes in an exposure. For each MR analysis, we removed SNPs missing from the outcome dataset as well as palindromic SNPs with intermediate allele frequencies. The strength of the eligible IVs was calculated by the F-statistic.

### Heterogeneity, pleiotropy and sensitivity analysis

In addition, Cochran’s Q test, which includes IVW, maximum likelihood, and MR‒Egger, was further applied to assess heterogeneity among the effects of individual COVID-19 infection or hospitalization-associated SNPs on the mRNFL and mGCIPL. A P > 0.05 indicated that there was no heterogeneity in the included IVs, and the effect of heterogeneity on the estimation of causal effects could be ignored. MR‒Egger regression and the MR pleiotropy RESidual Sum and Outlier (PRESSO) test were performed to further identify potential directional pleiotropy, and a P > 0.05 indicated a lack of horizontal pleiotropy [17]. In addition, we applied variants of the IVW model to better visualize the causal estimate.

Sensitivity analysis was also conducted to verify the MR model assumptions and evaluate the MR results. Leave-one-out analysis was also used to assess potentially influential SNPs and verify the reliability of the MR estimates. For each SNP, a causal effect was generated for relative outcomes as ORs per standard deviation unit increase in the putative risk factor using the Wald ratio. All the statistical analyses in this study were conducted using the “TwoSampleMR” package and the MR-PRESSO R package in R software (version 4.2.1). All presented P values were two-sided, and statistical significance was set at the 5% level.

## Results

### Results of COVID-19 infection and hospitalization-associated SNPs

In our MR analysis, 24 COVID-19 infection-associated SNPs and 42 COVID-19 hospitalization-associated SNPs were selected as IVs for the outcome GWAS of mRNFL and mGCIPL (Additional file 2: Table S1). Then, we searched for the selected SNPs in the PhenoScanner dataset and identified any potential SNPs associated with other confounding factors. SNPs that did not pass the identification were deleted by setting a proxy. After harmonization and removal of palindromic SNPs with intermediate allele frequencies, 22 and 40 eligible SNPs were considered IVs for COVID-19 infection and COVID-19 hospitalization, respectively. The F-statistics for IVs of COVID-19 infection and COVID-19 hospitalization were greater than 10 (range: 29.73–462.59 and 29.95–968.94, respectively), indicating that these SNPs were strong enough for MR analysis (Additional file 2: Table S1).

### COVID-19 infection was associated with an increased risk of mRNFL and mGCIPL thickness, whereas COVID-19 hospitalization was not

**Fig. 2:**
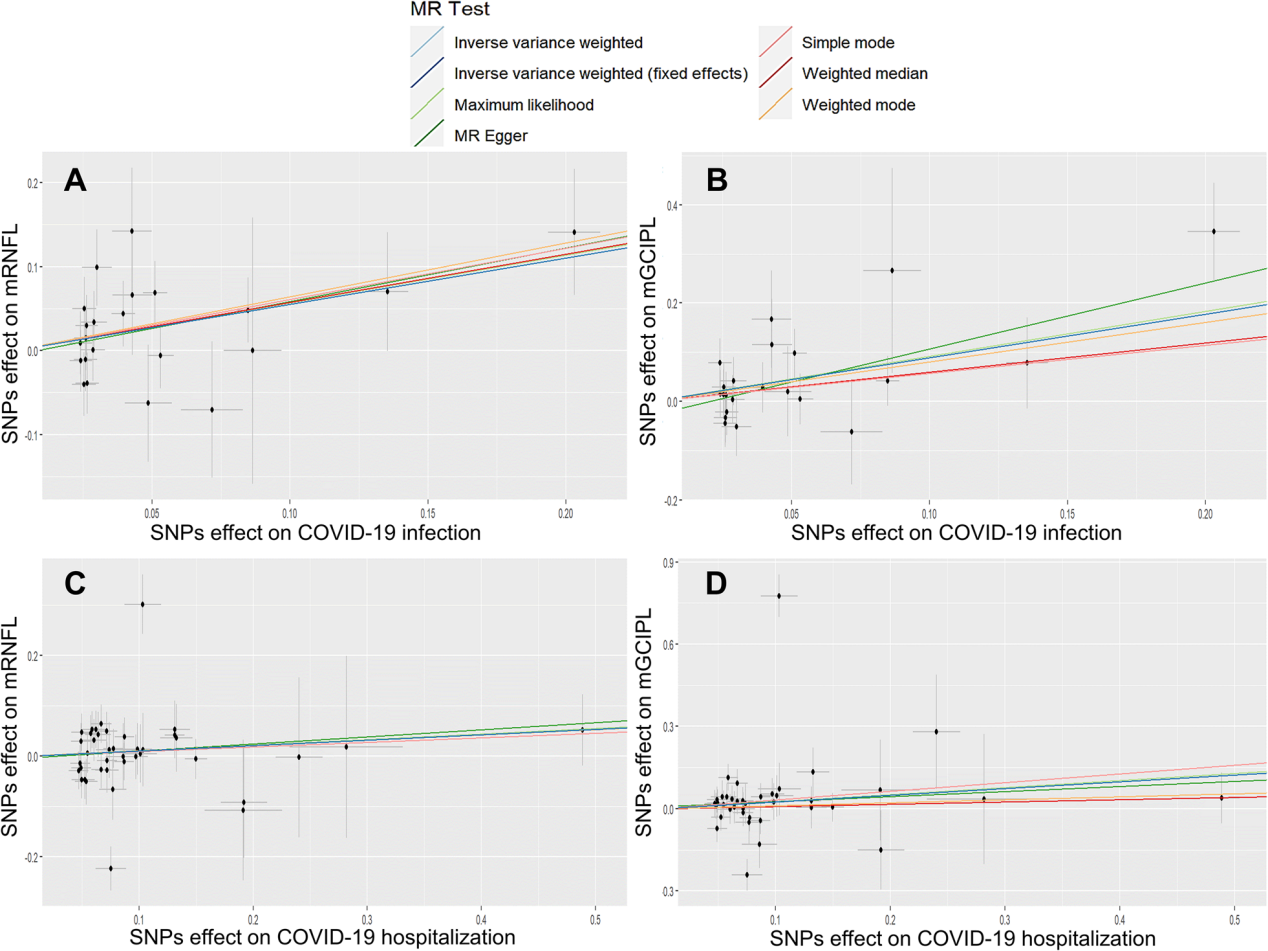
Scatter plots of MR analyses from COVID-19 infection or hospitalization to mRNFL or mGCIPL in each database. **A**, **B** The causal relationship between COVID-19 infection and mRNFL or mGCIPL. **C**, **D** The causal relationship between COVID-19 hospitalization and mRNFL or mGCIPL. Black dots denote the genetic instruments included in the MR analyses

The MR analysis of the effects of exposure (COVID-19 infection and hospitalization) on outcomes (mRNFL and mGCIPL) are shown in Tables 1, 2. According to Table 1, there was no obvious evidence of heterogeneity in the association between COVID-19 infection-associated SNPs and the mRNFL or mGCIPL (P = 0.61 and 0.69, respectively). The IVW results demonstrated that genetic-predicted COVID-19 infection significantly increased both the thickness of the mRNFL and the thickness of the mGCIPL (OR = 1.74, 95% CI 1.20–2.52, P = 3.60 × 10^–3^; OR = 2.43, 95% CI 1.49–3.96, P = 3.6 × 10^–4^). However, the results in Table 2 show that COVID-19-related hospitalization did not increase the thickness of the mRNFL or mGCIPL (OR = 1.11, 95% CI 0.90–1.37, P = 0.32; OR = 1.28, 95% CI 0.88–1.85, P = 0.19), with significant heterogeneity (P = 1.91 × 10^–4^, 1.27 × 10^–13^). Similar effect estimates were also obtained through the maximum likelihood-based and MR-PRESSO methods, which enhanced the robustness of the causal associations. Scatter plots of MR data from patients with COVID-19 infection or hospitalization to the mRNFL or mGCIPL in each database provided us with a better visualization of the association between SARS-CoV-2 and the inner retina (Fig. 2).

**Table 1:** The MR analysis, heterogeneity and pleiotropy of COVID-19 infection on mRNFL, mGCIPL thickness

Method	Outcome	Exposure: COVID-19 infection						
		N	Effect (SE)	P	OR	95% CI	P-heterogeneity	P-pleiotropy
IVW	mRNFL	22	0.19	3.6 × 10^–3^	1.74	1.20–2.52	0.61	
Maximum likelihood		22	0.19	2.82 × 10^–3^	1.77	1.22–2.58	0.61	
MR Egger		22	0.32	0.06	1.89	1.01–3.55	0.55	
IVW (fixed effects)		22	0.19	3.58 × 10^–3^	1.74	1.20–2.52	–	
MR PRESSO		22	0.18	0.26	1.19	1.17–2.34	–	0.74
IVW	mGCIPL	22	0.25	3.60 × 10^–4^	2.43	1.49–3.96	0.69	
Maximum likelihood		22	0.25	2.50 × 10^–3^	2.50	1.53–4.08	0.70	
MR Egger		22	0.42	4.62 × 10^–3^	3.83	1.68–8.75	0.74	
IVW (fixed effects)		22	0.25	3.60 × 10^–4^	2.43	1.49–3.96	**–**	
MR PRESSO		22	0.25	0.60	1.29	1.27–3.43	**–**	0.19

*MR* Mendelian randomization, *N* number of SNPs used in MR analysis, *OR* odds ratio, *CI* confidence interval, *IVW* inverse variance weighted, *PRESSO* pleiotropy residual sum and outlier

**Table 2:** The MR analysis, heterogeneity and pleiotropy of COVID-19 hospitalization on mRNFL or mGCIPL thickness

Method	Outcome	Exposure: COVID-19 hospitalization						
		N	Effect (SE)	P	OR	95% CI	P-heterogeneity	P-pleiotropy
IVW	mRNFL	40	0.11	0.32	1.11	0.90–1.37	1.91 × 10^–4^	
Maximum likelihood		40	0.08	0.15	1.12	0.96–1.30	1.94 × 10^–4^	
MR Egger		40	0.20	0.49	1.15	0.78–1.70	1.32 × 10^–4^	
IVW (fixed effects)		40	0.08	0.16	1.11	0.96–1.29	–	
MR PRESSO		40	0.11	0.94	1.11	0.92–1.41	–	0.84
IVW	mGCIPL	40	0.19	0.19	1.28	0.88–1.85	1.27 × 10^–13^	
Maximum likelihood		40	0.10	0.01	1.29	1.06–1.58	1.37 × 10^–13^	
MR Egger		40	0.20	0.60	1.20	0.60–2.41	6.82 × 10^–14^	
IVW (fixed effects)		40	0.08	0.01	1.28	1.05–1.55	–	
MR PRESSO		40	0.19	0.24	1.20	0.90–1.85	–	0.84

*MR* Mendelian randomization, *N* number of SNPs used in MR analysis, *OR* odds ratio, *CI* confidence interval, *IVW* inverse variance weighted, *PRESSO* pleiotropy residual sum and outlier

### Results of pleiotropy and sensitivity analysis

**Fig. 3:**
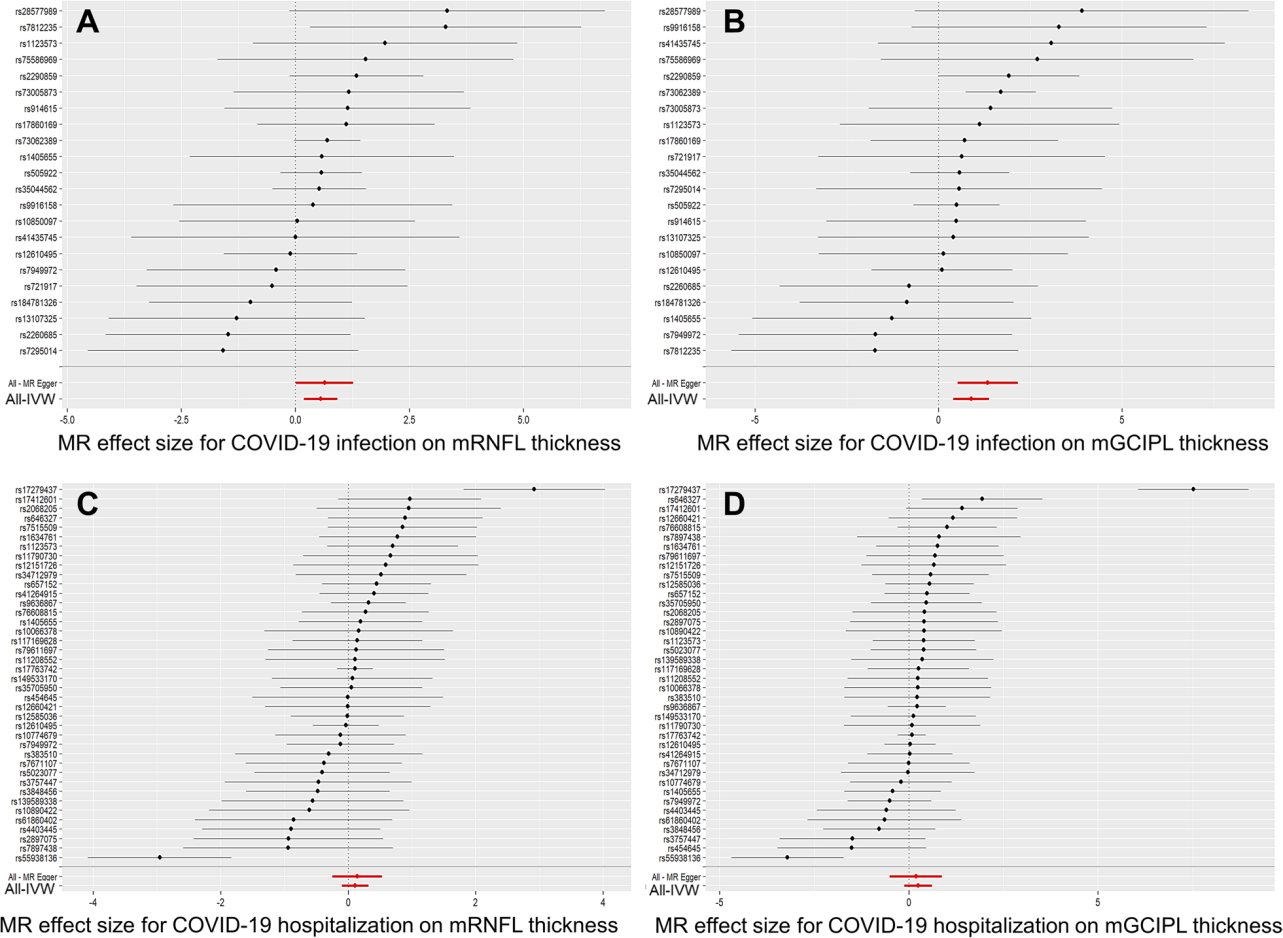
Forest plot of MR analyses from COVID-19 infection (**A**, **B**) or hospitalization (**C**, **D**) to mRNFL and mGCIPL in each database

As shown in Tables 1, 2, MR‒Egger regression of COVID-19 infection on the mRNFL and mGCIPL showed no evidence of targeted polymorphisms (P = 0.74 and 0.19, respectively) or of COVID-19 hospitalization (P = 0.84 and 0.84, respectively). According to the sensitivity analyses, the forest plots and the leave-one-out sensitivity analysis also demonstrated that causal links between genetically determined COVID-19 infection and COVID-19 hospitalization according to the mRNFL or mGCIPL were not driven by any single SNP, suggesting the stability of our MR analysis **(**Fig. 3 and the Additional file 1: Fig. S1).

## Discussion

The results of the present study allowed us to evaluate the effects of SARS-CoV-2 on the inner retina at the genetic level for the first time, filling the gap in the literature in which OCT could not be performed under isolated conditions after infection. Our results showed that the genetically predicted thickness of the inner retina was significantly increased by SARS-CoV-2 infection.

Previous studies have pointed to retinal involvement in COVID-19, such as central retinal artery/vein occlusion and acute macular neuroretinopathy, which can directly damage vision [10, 11, 18]. RGCs are particularly susceptible to SARS-CoV-2 infection, which can directly cause acute pathological changes and increase overall retinal thickness due to immune cells infiltration [19, 20]. Mechanistically, the viral infection induces a procoagulant state and endothelial inflammation with recruitment of inflammatory cells, resulting in micro-thrombosis and retinal thickening in the early stage [10, 21–23].

Our findings support the findings of previous studies. However, some observational studies hold different conclusions [21]. After reviewing the literature, the controversies may be due to the difference in the duration of acute infection and ophthalmic evaluation. As the duration of COVID-19 recovery varies widely, most observational studies have been performed in the subacute phase (4 weeks) after infection [24]. Confounding and detrimental variables such as hospitalization and steroid use could influence the results and may not represent the normal duration of the acute early phase of the disease [21].

Interestingly, evidence from our MR analysis suggested that the mRNFL and mGCIPL thicknesses did not significantly differ between hospitalized COVID-19 patients and healthy individuals. Similar findings have also been reported in previous studies. For example, OCT measurements of hospitalized COVID-19 patients revealed no alterations in retinal thickness after treatment with famipiravir, moxifloxacin, or heparin [25]. In addition, steroid administration significantly improved the condition of a COVID-19 patient who developed severe monocular vision loss with white dot outer retinopathy [26]. The main underlying mechanism of COVID-19 is active viral replication followed by a dysregulated immune response and systemic hyperinflammation [27]. Therefore, reducing viral replication in combination with anti-inflammatory treatment may be more helpful in the early stage.

As COVID-19 treatments approved in the European Union and according to clinical recommendations, non-hospitalized patients with mild to moderate COVID-19 infection are usually treated symptomatically [27]. However, additionally, hospitalized patients with severe COVID-19 would be administered systemic corticosteroids and low-molecular heparin for anti-inflammation and anticoagulation treatment, as well as supportive respiratory care [28]. Importantly, the treatment modalities may vary regionally, but they are generally similar. Together with our findings, it seems possible that due to differences in treatment between COVID-19 infection and hospitalized patients, the genetic prediction of inner retina thickness by MR analysis was completely different. Therefore, we speculated that some of the treatments administered to hospitalized COVID-19 patients may reverse the increase in retinal thickness, which would not only be effective at preventing potential visual complications but also provide new insights into the current management of SARS-CoV-2 patients. However, it should be noted that the MR analysis of hospitalized COVID-19 patients exhibited a high degree of heterogeneity. This may be due to differences in treatment modalities, as previously mentioned.

The late stage of SARS-CoV-2 infection is thought to be associated with apoptosis and retinal degeneration, potentially leading to atrophy [29]. However, the long-term effects of SARS-CoV-2, which has known as “long COVID,” are also a concern worldwide. As a neurophilic virus, SARS-CoV-2 can infect and replicate in retinal and brain organoids, resulting in neurodegenerative disease in susceptible individuals in the long run [30].

It was reported that most cases of the long COVIDs were non-hospitalized patients with a mild-acute illness [30]. In the eyes, long COVID was found to include loss of small corneal nerve fibers, increased dendritic cell density, and significantly impaired retinal microcirculation [30, 31]. Furthermore, several reports have indicated that postrecovery SARS-CoV-2 patients have a significantly thinner RNFL than healthy individuals [18, 32, 33]. And a reduced RNFL thickness has been linked to an increased risk of various diseases, such as Alzheimer's disease and glaucoma [34, 35].

There is still a lot we do not know about long COVID. It should be noted that visual impairment and ultimately blindness from retinal degenerative disorders may become apparent only after a protracted course of development. However, it is possible to minimize the incidence of this disease with appropriate treatments. Therefore, taken together, emphasis on the prevention of damage in the acute phase is crucial and urgent. In the absence of absolute contraindications, indications for the use of some drugs, such as steroids and low-molecular heparin, are likely to be moderately relaxed in the future. This could also potentially alleviate some of the medical burden.

Compared with traditional observational studies, the greatest advantage of this study is that the causal estimate obtained by MR avoids reverse causality and confounding bias. Additionally, applying comprehensive GWAS data for MR analyses can improve the accuracy of the estimated effect [36]. However, several limitations cannot be avoided in our study. First, as mentioned above, changes in retinal findings cannot be assessed over time due to the SARS-CoV-2 pandemic, and additional studies are needed to determine the longitudinal evolution of retinal changes over time. Second, to reduce the risk of population stratification, the MR analyses were only for Europeans, data for other ethnic groups were lacking; moreover, due to the lack of GWAS datasets, we did not perform sex or age stratification analysis. Third, we investigated the linear relationship between SARS-CoV-2 and retinal phenotypes but cannot rule out the possibility of a nonlinear effect.

## Conclusion

In conclusion, we found genetic evidence supporting a causal association between COVID-19 infection and increased thickness of the inner retina. In contrast, retinal thickness was not affected in hospitalized COVID-19 patients. However, this could be another indication that hospitalization interventions are effective. We still need to be vigilant in potential retinopathy as a sequela of long COVID, especially paying attention to retinas that are already unhealthy and watch for fundus abnormalities.

### Supplementary Information

**Additional file 1: Figure S1.** Leave-one-out plot of MR analyses from COVID-19 infection (**A**, **B**) or hospitalization (**C**, **D**) on mRNFL and mGCIPL in each database.

**Additional file 2: Table S1A.** Summary of genetic variants (n=24) used to estimate the effect of COVID-19 infection on inner retina in MR analyses. **Table S1B.** Summary of genetic variants (n=42) used to estimate the effect of COVID-19 hospitalization on inner retina in MR analyses.

## Data Availability

The data were publicly accessible, and data generated or analyzed during this study are included in this published article or in the data repositories listed in the references.
