# Supplementary material for: SARS-CoV-2 neurovascular invasion supported by Mendelian randomization
Source: J Transl Med. 2024 Jan 24;22:101. doi: 10.1186/s12967-024-04907-3 (PMC10809787; doi:10.1186/s12967-024-04907-3)
Supplement: Supplementary file 1 — Additional file 1: Figure S1. Leave-one-out plot of MR analyses from COVID-19 infection (A, B) or hospitalization (C, D) on mRNFL and mGCIPL in each database. [file 12967_2024_4907_MOESM1_ESM.docx]

**
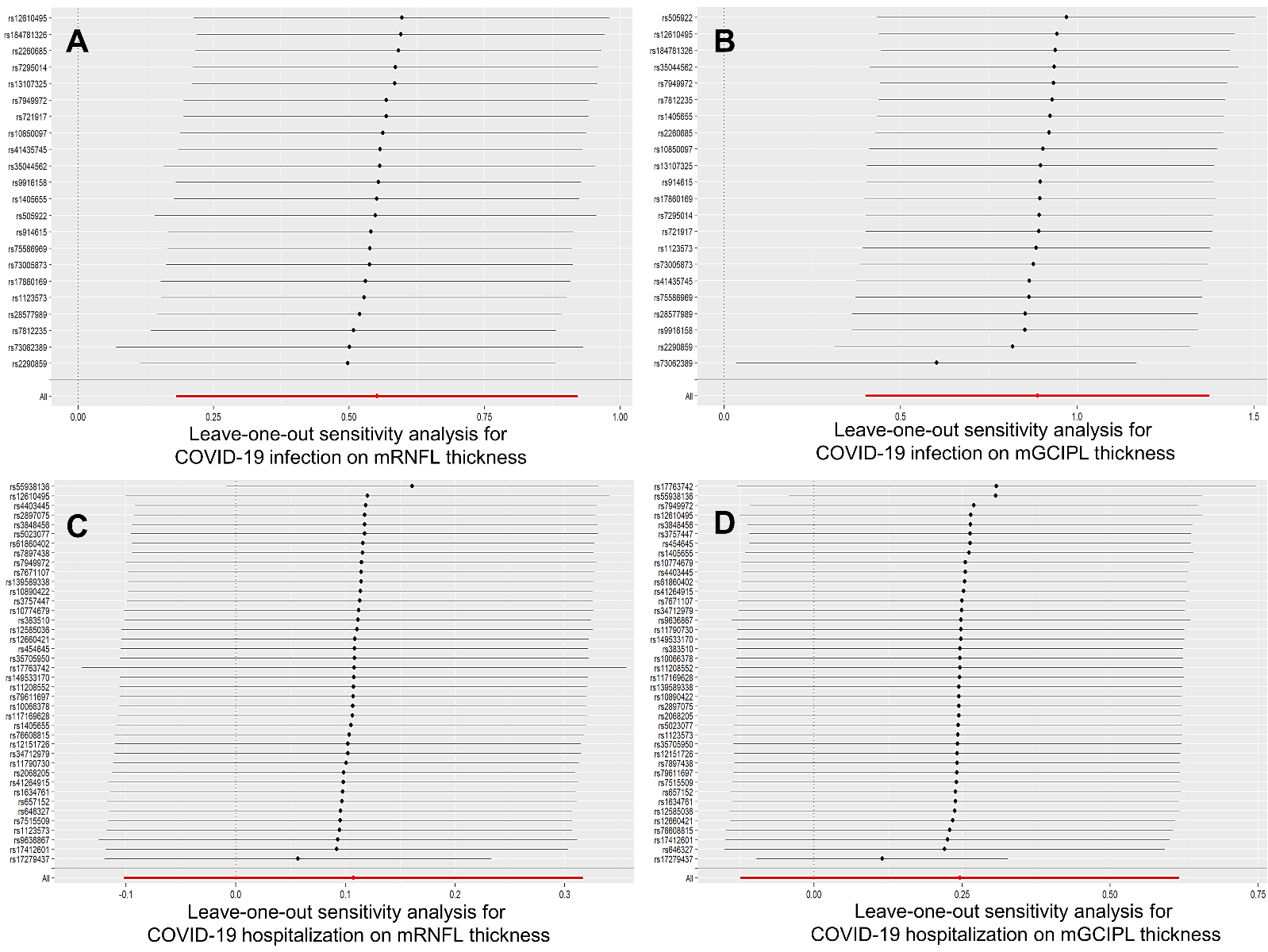
**

**Figure S1.** Leave-one-out plot of MR analyses from COVID-19 infection (**AB**) or hospitalization (**CD**) on mRNFL and mGCIPL in each database.
