# Supplementary material for: SARS-CoV-2 neurovascular invasion supported by Mendelian randomization
Source: J Transl Med. 2024 Jan 24;22:101. doi: 10.1186/s12967-024-04907-3 (PMC10809787; doi:10.1186/s12967-024-04907-3)
Supplement: Supplementary file 2 — Additional file 2: Table S1A. Summary of genetic variants (n=24) used to estimate the effect of COVID-19 infection on inner retina in MR analyses. Table S1B. Summary of genetic variants (n=42) used to estimate the effect of COVID-19 hospitalization on inner retina in MR analyses. [file 12967_2024_4907_MOESM2_ESM.docx]

**Table S1A.** Summary of genetic variants (n=24) used to estimate the effect of COVID-19 infection on inner retina in MR analyses.

| **No.** | **SNP** | **Chr.** | **Position** | **EA** | **OA** | **EAF** | **Beta** | **SE** | **P-Value** | **F-Statistic** |
| --- | --- | --- | --- | --- | --- | --- | --- | --- | --- | --- |
| 1 | rs10850097 | 12 | 113361117 | T | C | 0.6789 | 0.028587 | 0.00436 | 5.5004E-11 | 42.98968 |
| 2 | rs1123573 | 2 | 60707588 | G | A | 0.3668 | -0.02539 | 0.004332 | 4.617E-09 | 34.34519 |
| 3 | rs12610495 | 19 | 4717672 | G | A | 0.2907 | 0.053077 | 0.00473 | 3.212E-29 | 125.9134 |
| 4 | rs13107325 | 4 | 103188709 | T | C | 0.05551 | 0.048611 | 0.008522 | 1.1702E-08 | 32.53535 |
| 5 | rs1405655 | 19 | 50882619 | C | T | 0.3322 | 0.026014 | 0.004279 | 1.2024E-09 | 36.96672 |
| 6 | rs17860169 | 21 | 34613301 | G | A | 0.3374 | 0.039565 | 0.004325 | 5.76E-20 | 83.70096 |
| 7 | rs184781326 | 14 | 29459234 | G | A | 0.04315 | -0.07169 | 0.011215 | 1.6282E-10 | 40.86646 |
| 8 | rs2260685 | 3 | 195497743 | C | T | 0.4625 | 0.026488 | 0.004238 | 4.084E-10 | 39.07319 |
| 9 | rs2290859 | 3 | 101525625 | T | C | 0.3413 | -0.0511 | 0.004407 | 4.4262E-31 | 134.4221 |
| 10 | rs28577989 | 6 | 31255762 | C | A | 0.08818 | 0.042704 | 0.0072 | 3.0123E-09 | 35.17612 |
| 11 | rs35044562 | 3 | 45909024 | G | A | 0.07729 | 0.13537 | 0.007549 | 6.5644E-72 | 321.563 |
| 12 | rs41435745 | 6 | 41490382 | C | G | 0.04448 | 0.086439 | 0.010481 | 1.6248E-16 | 68.01646 |
| 13 | rs505922 | 9 | 136149229 | T | C | 0.6516 | -0.08478 | 0.004219 | 7.9282E-90 | 403.8586 |
| 14 | rs679574 | 19 | 49206108 | G | C | 0.4389 | -0.03646 | 0.004167 | 2.1403E-18 | 76.55725 |
| 15 | rs721917 | 10 | 81706324 | G | A | 0.4294 | 0.024172 | 0.004097 | 3.6473E-09 | 34.80404 |
| 16 | rs7295014 | 12 | 133067989 | A | G | 0.6386 | 0.025314 | 0.004311 | 4.2902E-09 | 34.48786 |
| 17 | rs73005873 | 19 | 9007630 | A | G | 0.3645 | 0.028961 | 0.004688 | 6.5311E-10 | 38.15731 |
| 18 | rs73062389 | 3 | 45835417 | A | G | 0.05157 | 0.20302 | 0.009439 | 1.3093E-102 | 462.5919 |
| 19 | rs75586969 | 21 | 35265459 | T | C | 0.08208 | 0.042867 | 0.007212 | 2.781E-09 | 35.33124 |
| 20 | rs7812235 | 7 | 100211486 | C | G | 0.2006 | -0.03003 | 0.005403 | 2.7181E-08 | 30.89803 |
| 21 | rs7949972 | 11 | 34502042 | T | C | 0.3562 | -0.02586 | 0.004209 | 8.0042E-10 | 37.75901 |
| 22 | rs914615 | 1 | 155175892 | A | G | 0.451 | 0.026293 | 0.004088 | 1.2578E-10 | 41.37147 |
| 23 | rs9519214 | 13 | 88691699 | T | A | 0.455 | 0.039336 | 0.007214 | 4.9628E-08 | 29.73062 |
| 24 | rs9916158 | 17 | 38182229 | T | G | 0.365 | 0.02404 | 0.004208 | 1.1083E-08 | 32.64065 |
|  |  |  |  |  |  |  |  |  |  |  |
| Abbreviations: SNP, single-nucleotide polymorphism; Chr, chromosome; EA, effect allele; OA, other allele; EAF, effect allele frequency; SE, standard error. | | | | | | | | | | |

**Table S1B.** Summary of genetic variants (n=42) used to estimate the effect of COVID-19 hospitalization on inner retina in MR analyses.

| **No.** | **SNP** | **Chr.** | **Position** | **EA** | **OA** | **EAF** | **Beta** | **SE** | **P-Value** | **F-Statistic** |
| --- | --- | --- | --- | --- | --- | --- | --- | --- | --- | --- |
| 1 | rs10066378 | 5 | 131776967 | C | T | 0.1324 | 0.074001 | 0.011575 | 1.63E-10 | 40.87269 |
| 2 | rs10774679 | 12 | 113374748 | T | C | 0.6527 | 0.071847 | 0.008801 | 3.25E-16 | 66.64431 |
| 3 | rs10890422 | 1 | 47275770 | C | T | 0.4054 | -0.04717 | 0.008619 | 4.43E-08 | 29.95203 |
| 4 | rs11208552 | 1 | 65412830 | T | G | 0.6393 | -0.05498 | 0.009189 | 2.19E-09 | 35.79344 |
| 5 | rs1123573 | 2 | 60707588 | G | A | 0.3645 | -0.07171 | 0.009308 | 1.32E-14 | 59.34782 |
| 6 | rs117169628 | 16 | 89262657 | A | G | 0.133 | 0.098534 | 0.012798 | 1.37E-14 | 59.27725 |
| 7 | rs11790730 | 9 | 33425785 | C | T | 0.1932 | 0.064167 | 0.011156 | 8.82E-09 | 33.08312 |
| 8 | rs12151726 | 2 | 198273591 | T | C | 0.4049 | 0.049296 | 0.008976 | 3.97E-08 | 30.16519 |
| 9 | rs12585036 | 13 | 113535741 | T | C | 0.2076 | 0.097378 | 0.010296 | 3.15E-21 | 89.45088 |
| 10 | rs12610495 | 19 | 4717672 | G | A | 0.2907 | 0.1499 | 0.009868 | 4.06E-52 | 230.7751 |
| 11 | rs12660421 | 6 | 41488378 | A | G | 0.04235 | 0.24033 | 0.02066 | 2.82E-31 | 135.3179 |
| 12 | rs139589338 | 1 | 154826289 | G | A | 0.01888 | 0.19135 | 0.033938 | 1.72E-08 | 31.78955 |
| 13 | rs1405655 | 19 | 50882619 | C | T | 0.3326 | 0.077686 | 0.008735 | 5.92E-19 | 79.09332 |
| 14 | rs149533170 | 9 | 21172825 | A | G | 0.007759 | 0.28165 | 0.049626 | 1.38E-08 | 32.21076 |
| 15 | rs1634761 | 6 | 31274027 | T | C | 0.5009 | -0.05761 | 0.008324 | 4.50E-12 | 47.89113 |
| 16 | rs17279437 | 3 | 45814094 | A | G | 0.09658 | -0.10328 | 0.016004 | 1.09E-10 | 41.6462 |
| 17 | rs17412601 | 3 | 101499275 | C | T | 0.3408 | -0.06678 | 0.009044 | 1.54E-13 | 54.51624 |
| 18 | rs17763742 | 3 | 45846769 | G | A | 0.07814 | 0.48852 | 0.015694 | 1.00E-200 | 968.9411 |
| 19 | rs2068205 | 6 | 33058583 | C | T | 0.6199 | 0.049693 | 0.008902 | 2.38E-08 | 31.15988 |
| 20 | rs2569703 | 19 | 10404227 | G | C | 0.542 | -0.05456 | 0.008333 | 5.85E-11 | 42.87083 |
| 21 | rs2897075 | 7 | 99630342 | T | C | 0.3648 | 0.049692 | 0.008642 | 8.92E-09 | 33.06393 |
| 22 | rs34712979 | 4 | 106819053 | A | G | 0.2336 | -0.06072 | 0.011036 | 3.76E-08 | 30.27193 |
| 23 | rs35705950 | 11 | 1241221 | T | G | 0.09965 | -0.10119 | 0.01454 | 3.41E-12 | 48.43356 |
| 24 | rs3757447 | 7 | 22894735 | T | A | 0.5914 | -0.04929 | 0.008748 | 1.75E-08 | 31.75067 |
| 25 | rs383510 | 21 | 42858367 | C | T | 0.5429 | -0.04846 | 0.008726 | 2.79E-08 | 30.8468 |
| 26 | rs3848456 | 17 | 47940623 | A | C | 0.03875 | 0.19199 | 0.020254 | 2.56E-21 | 89.85363 |
| 27 | rs41264915 | 1 | 155167786 | G | A | 0.1035 | -0.13127 | 0.013369 | 9.33E-23 | 96.41245 |
| 28 | rs4403445 | 8 | 61432007 | A | G | 0.396 | 0.05261 | 0.008423 | 4.21E-10 | 39.01243 |
| 29 | rs454645 | 3 | 146232469 | T | C | 0.0743 | 0.086188 | 0.015314 | 1.82E-08 | 31.67498 |
| 30 | rs5023077 | 12 | 133141973 | C | T | 0.5167 | -0.06675 | 0.008451 | 2.84E-15 | 62.3725 |
| 31 | rs55938136 | 17 | 43798360 | G | A | 0.1901 | -0.0754 | 0.013335 | 1.57E-08 | 31.97018 |
| 32 | rs61860402 | 10 | 81723695 | T | C | 0.113 | 0.076982 | 0.011995 | 1.38E-10 | 41.18868 |
| 33 | rs646327 | 19 | 49209851 | G | A | 0.4862 | -0.05886 | 0.008626 | 8.90E-12 | 46.55884 |
| 34 | rs657152 | 9 | 136139265 | C | A | 0.6111 | -0.08735 | 0.008485 | 7.51E-25 | 105.9648 |
| 35 | rs732990 | 2 | 26842146 | G | C | 0.5149 | 0.051356 | 0.008871 | 7.07E-09 | 33.51485 |
| 36 | rs7515509 | 1 | 77949123 | A | G | 0.3681 | 0.062296 | 0.008924 | 2.94E-12 | 48.72726 |
| 37 | rs76608815 | 21 | 35353264 | T | C | 0.08976 | 0.13283 | 0.014161 | 6.60E-21 | 87.98416 |
| 38 | rs7671107 | 4 | 25449225 | A | G | 0.7314 | -0.07189 | 0.010204 | 1.84E-12 | 49.63868 |
| 39 | rs7897438 | 10 | 114732307 | A | C | 0.2409 | -0.05374 | 0.009847 | 4.83E-08 | 29.78209 |
| 40 | rs7949972 | 11 | 34502042 | T | C | 0.3545 | -0.08701 | 0.008542 | 2.28E-24 | 103.7646 |
| 41 | rs79611697 | 9 | 15795833 | T | G | 0.0692 | 0.10347 | 0.018776 | 3.57E-08 | 30.36846 |
| 42 | rs9636867 | 21 | 34609944 | G | A | 0.3378 | 0.13121 | 0.008773 | 1.41E-50 | 223.7007 |
|  | | | | | | | | | | |
| Abbreviations: SNP, single-nucleotide polymorphism; Chr, chromosome; EA, effect allele; OA, other allele; EAF, effect allele frequency; SE, standard error. | | | | | | | | | | |
